# Interventions for preventing, delaying the onset, or decreasing the burden of frailty: an overview of systematic reviews

**DOI:** 10.1186/s13643-015-0110-7

**Published:** 2015-09-25

**Authors:** Michael G. Wilson, François Béland, Dominic Julien, Lise Gauvin, G. Emmanuel Guindon, Denis Roy, Kaitryn Campbell, Donna G. Comeau, Heather Davidson, Parminder Raina, Deborah Sattler, Brenda Vrkljan

**Affiliations:** McMaster Health Forum, McMaster University, Hamilton, Canada; Centre for Health Economics and Policy Analysis, McMaster University, Hamilton, Canada; Department of Clinical Epidemiology and Biostatistics, McMaster University, Hamilton, Canada; Département d’administration de la santé, École de santé publique, Université de Montréal, Montréal, Canada; Institut Lady Davis, Hôpital général juif, Montréal, Canada; Centre de recherche de l’Institut universitaire en santé mentale de Montréal, Université de Montréal, Montréal, Canada; Département de psychologie, Université de Montréal, Montréal, Canada; Department of Social and Preventive Medicine at the Université de Montréal, Montréal, Canada; l’Institut National d’Excellence en Santé et en Services Sociaux, Québec, Canada; Programs for Assessment of Technology in Health (PATH), McMaster University, Hamilton, Canada; Department of Health and Wellness, Halifax, NS Canada; McMaster Evidence Review and Synthesis Centre, McMaster University, Hamilton, Canada; British Columbia Ministry of Health, Victoria, Canada; Erie St. Clair Local Health Integration Network, Chatham, Canada; School of Rehabilitation Science, McMaster University, Hamilton, Canada

**Keywords:** Frailty, Ageing, Seniors, Older adults, Interventions, Overview of systematic reviews, Stakeholder dialogue

## Abstract

**Background:**

Many systematic reviews have evaluated the effectiveness of interventions to prevent, delay, or decrease frailty symptoms, but no effort has been made to identify, map, and synthesize the findings from reviews across the full spectrum of interventions. Our objectives are to (1) synthesize findings from all existing systematic reviews evaluating interventions for preventing, delaying the onset, or decreasing the burden of frailty symptoms; (2) examine different conceptualizations of frailty that have been used in the development and implementation of interventions; and (3) inform policy by convening a stakeholder dialogue with Canadian health-system leaders.

**Methods/design:**

We will conduct an overview of systematic reviews to identify and synthesize all of the systematic reviews addressing interventions to preventing, delaying the onset, or decreasing the burden of frailty symptoms. To identify relevant systematic reviews, we will conduct database searches for published and grey literature as well as contact key experts and search reference lists of included reviews. Two reviewers will independently review all search results for inclusion and then conceptually map, extract key findings (including the conceptualization/definition of frailty used) and assess the methodological quality of all included reviews. We will then synthesize the findings by producing a ‘gap map’ (i.e. mapping reviews in a matrix according to the interventions and outcomes assessed), and narratively synthesize the key messages across reviews related to type of interventions.

**Discussion:**

Following the completion of the synthesis, we will use the findings to develop an evidence brief that mobilizes the best available evidence about the problem related to preventing, delaying the onset, or decreasing the burden of frailty symptoms in older adults, policy and programmatic options to address the problem and implementation considerations. The evidence brief will then be used as the input into a stakeholder dialogue, which will engage 18–22 Canadian health-system leaders (including policymakers, health providers, researchers, and other stakeholders) in ‘off-the-record’ deliberations to inform future actions and policymaking.

**Systematic review registration:**

PROSPERO CRD42015022082

**Electronic supplementary material:**

The online version of this article (doi:10.1186/s13643-015-0110-7) contains supplementary material, which is available to authorized users.

## Background

In general frailty refers to ‘older adults or aged individuals who are lacking in general strength and are unusually susceptible to disease or to other infirmity’ [1]. However, a diversity of conceptualizations of frailty have been proposed in the literature, ranging from a physical performance model [2] to a multidimensional model [3, 4] through conceptualizations of frailty as a medical syndrome [5, 6], a geriatric syndrome [7], or as deficit accumulation [8]. Also, a systematic review examined operational definitions of frailty and identified 15 components in the different conceptualizations and screening tools for frailty (e.g. physical function, mobility, cognition) that were included in the review [9]. Given the lack of consensus on a definition of frailty as well as criteria to identify or ‘diagnose’ frailty, estimates of prevalence are limited and highly variable, ranging from 4–59 % depending on the population being studied [5, 10–15]. Regardless, the number of frail older adults will increase as the population continues to age [6, 16], which has important implications for health systems given that those who are frail have greater risks of disabilities in basic and instrumental activities of daily living [17, 18], chronic illnesses [17, 19], greater reliance on in-home services [20], hospitalization [20, 21], institutionalization [22, 23], and premature mortality [23–25]. Therefore, as a result of population ageing, adverse consequences of frailty, and large social costs and burden for families and caregivers, there is a need to identify effective interventions to prevent or delay the onset as well as decrease the burden of frailty symptoms among older adults. Aiming such efforts at both pre-frail and frail older adults represents an opportunity for preventing or delaying the onset of frailty for those most at risk, improving the effectiveness of prevention and the delivery of care, and improving the health and quality of life for individuals with complex health needs.

Previous reviews have focused on interventions targeting frailty, but they address specific interventions, such as screening tools [26], home-based support [27], home telecare [28], hospital discharge planning [29], physical activity programmes [30], or health promotion [31]. Although these specific interventions are important individually, addressing frailty likely requires a comprehensive approach involving coordination across a broad range of interventions at clinical, public health, and system levels. To our knowledge, no reviews have synthesized findings across the entire spectrum of interventions aimed at pre-frail and frail older persons. In addition to this gap, there is a lack of consensus on what constitutes frailty due to a broad range of conceptualizations that are currently used [16, 32–40]. It is unclear whether the different conceptualizations (e.g. syndrome vs. accumulation deficit) are associated with different intervention strategies (e.g. enhancing physical capacities vs. coping strategies).

### Objectives

Our objectives are designed to address these gaps by the following:

1. Synthesizing findings from all existing systematic reviews evaluating interventions for preventing, delaying the onset, or decreasing the burden of frailty symptoms (in accordance with how it is defined) among older adults
2. Examining different conceptualizations of frailty that have been used in the development and implementation of interventions, and their associations with these intervention strategies
3. Informing policymaking by convening a stakeholder dialogue with Canadian health-system leaders

## Methods/design

To achieve these objectives, we will conduct an overview of systematic reviews. We describe below our methods for conducting literature searches, reviewing search results, conceptual mapping, data extraction, quality appraisal, and synthesis.

### Literature searches

Our comprehensive search approach consists of three strategies for identifying published and unpublished literature. The strategy was developed by a trained library scientist (KC) and was peer reviewed by a library scientist at the McMaster Evidence Review and Synthesis Centre using the Peer Review of Electronic Search Strategies (PRESS) Checklist [41]. The first component of our strategy focuses on searching relevant databases including the following: the Cochrane Library (including Cochrane Database of Systematic Reviews and reviews indexed in the Database of Abstracts of Reviews of Effects), Medline, Embase, CINAHL, PsycINFO, EconLit, Social Sciences Abstracts and Applied Social Sciences Index and Abstracts (ASSIA), McMaster Optimal Aging Portal (a repository of reviews and primary research related to optimal ageing), HealthEvidence.org (for review related to public health), and Health Systems Evidence (a repository of systematic reviews addressing health-system topics). Our detailed search strategy is included in Additional file 1 and generally uses both controlled vocabulary, such as the National Library of Medicine’s MeSH (Medical Subject Headings), and keywords related to the topic of interest (e.g. frail*) with the population of interest (e.g. seniors, elderly, or older adults). In addition, for databases that index more than just systematic reviews, we combine these search terms with additional terms/filters that optimize the retrieval of systematic reviews. The second component of our strategy focuses on identifying grey literature by searching relevant databases (Open Grey and Grey Literature Report) as well as conducting targeted searches of websites/resources such as the Canadian Initiative on Frailty and Aging and the Canadian Geriatrics Society. We will supplement these searches by contacting key informants from local, national, and international organizations. Lastly, the third component of our search strategy will consist of handsearching reference lists of included reviews for other reviews meeting our inclusion criteria.

### Reviewing search results

Two pairs of team members (MGW and EG; FB and LG) pilot-tested draft inclusion criteria using 100 randomly selected references from our searches. Our final inclusion criteria require that (1) minimum criteria for a systematic review be met (explicit selection criteria and more than one database searched using an explicit and systematic search strategy); (2) the review explicitly focus, either partially or totally, on preventing, delaying the onset of or decreasing the burden of frailty (with ‘explicitly’ meaning that in the definition or description of the intervention(s), the words ‘frail’ or ‘frailty’ are mentioned); and (3) the focus of the review is on interventions for preventing, delaying the onset of or decreasing the burden of frailty. We provisionally define frailty as ‘older adults or aged individuals who are lacking in general strength and are unusually susceptible to disease or to other infirmity’ [1]. If a review is not limited to pre-frail or frail elders but also includes vulnerable or disabled elders, it must stratify results by category (e.g. pre-frail, frail, vulnerable, disabled) and provide clear conclusions about pre-frail and frail elders to be included.

The title and abstract of all search results will be reviewed in duplicate by two reviewers using the above inclusion criteria. The full text of each reference identified as ‘include’ or ‘unclear’ by at least one reviewer will undergo a final assessment by two independent reviewers for inclusion. At the stage of full-text reviewing, discordant conclusions will be resolved by consensus or by a third reviewer (MGW or FB) when consensus cannot be reached.

### Conceptual mapping

We will categorize reviews according to four domains (all categories included in the four domains are provided in Additional file 2). First, we will categorize reviews by sector based on a taxonomy of health-system arrangements used to organize documents indexed in Health Systems Evidence (www.healthsystemsevidence.org) [42]. The sectors in the taxonomy include primary care, home care, hospital care, rehabilitation, long-term care, and public health. Second, we will categorize reviews by type of provider(s) involved, which are derived from the same taxonomy and include physicians, nurses, nurse practitioners, pharmacists, allied health professionals, and lay/community health workers. Third, we will categorize the type of intervention(s) evaluated by grouping them according to those focused on prevention (e.g. identifying those at risk and health promotion activities), delaying the onset of frailty (e.g. rehabilitation for those who have suffered a fall that may lead to becoming frail), and decreasing the burden of frailty-related symptoms (based on the six domains of the Chronic Care Model) [43, 44]. Lastly, we will categorize reviews according to the types of outcomes included in the analysis using outcomes included within the Institute for Healthcare Improvement’s Triple Aim Initiative [45]. This initiative includes outcomes related to improving the patient experience of care, improving the health of populations, and reducing the per capita cost of care. We will iteratively refine this framework based on our increased familiarity with the range of interventions evaluated in the included systematic reviews.

### Extracting key findings

For each review, the following information will be extracted by one reviewer and independently checked for accuracy by a second reviewer: (1) focus of the review; (2) key messages (including positive and negative intervention effects); (3) conceptualization(s) of frailty used; (4) last year the literature was searched (as an indicator of how recent the findings of the review are); (5) population studied; and (6) countries in which studies included in the reviews were conducted. In extracting key findings, we will focus on identifying benefits, harms, and costs related to the intervention and list any potential barriers to implementation that are highlighted.

### Appraising quality

We will assess the quality of reporting of included systematic reviews using the AMSTAR (A Measurement Tool to Assess Systematic Reviews) tool [46]. AMSTAR ratings are already available for all reviews contained in Health Systems Evidence. For included reviews not from this source, the AMSTAR tool will be applied in duplicate by two reviewers. Any disagreements will be resolved by consensus and, in case a consensus could not be achieved, a third independent reviewer will resolve the disagreement.

### Synthesizing findings

We will narratively synthesize the results of our overview of systematic reviews, which we will then use to produce an evidence brief that will be used as the key input for a stakeholder dialogue (which is described in the ‘Discussion’ section). We will first use the results of the conceptual mapping to produce a ‘gap map’ using the approach developed by the International Initiative for Impact Evaluation [47]. This involves (1) developing a matrix with the intervention domains listed as the rows and the outcomes evaluated as the columns; (2) populating the cells with reviews based on the results of our conceptual mapping exercise; and (3) assessing the quality of reporting (high-, medium-, or low-quality) using colour-coding of the cells. Next, we will use the results of our data extraction to produce a distillation of the key messages related to each of the three intervention domains (preventing frailty, delaying the onset of frailty, and decreasing the burden of frailty-related symptoms) as well as a refined conceptualization of frailty. This will involve developing synthesis tables for each domain that outline the benefits, harms, and costs for the interventions identified and narratively summarizing the results. For reviews addressing the same topic and that include the same studies, we will focus on findings from reviews that were conducted most recently and with the highest quality of reporting and supplement with findings from other reviews where relevant. In addition, our analysis will involve thematically grouping the descriptions of frailty used in the reviews and conducting a content analysis to iteratively develop a refined conceptualization of the components and definitions of frailty. Each of these synthesis products will be supplemented with the detailed extraction tables, which will be included as appendices in a peer-reviewed manuscript. This process will be conducted by the scientific leads (MGW and FB), and iterations of the synthesis will be reviewed during regular meetings with our full team. To ensure transparency, we will follow the PRISMA (Preferred Reporting Items for Systematic reviews and Meta-Analyses) reporting guideline [48].

Following completion of the synthesis, the evidence brief will be developed using a multi-step process, which has been used successfully by the McMaster Health Forum to convene more than 30 stakeholder dialogues on pressing health-system issues (process and rationale for convening a dialogue are outlined in our discussion section below). This involves working in close collaboration with our research and knowledge-user partners to operationalize the findings from our overview of systematic reviews to (1) define the problem; (2) identify and describe what is known about possible options for addressing the problem; and (3) identify key implementation considerations for these options.

### Knowledge translation

Underlying our approach outlined above is an emphasis on stakeholder engagement as well as a multi-faceted approach to mobilizing our findings to inform policy and practice. For our commitment to stakeholder engagement, we have convened an interdisciplinary project team of researchers and knowledge users (including policymakers from four Canadian provinces). The team will meet regularly by teleconference to ensure the project is progressing as intended and to allow for the opportunity to collaboratively make project-related decisions. This will include (1) providing input at each stage of the project; (2) guiding the preparation of the ‘gap map’, synthesis, and evidence brief; (3) identifying participants for the stakeholder dialogue; (4) guiding the preparation of the dialogue summary led by the project leaders; and (5) preparing documentation for personalized interactive briefings following the stakeholder dialogue.

As the centrepiece of our knowledge translation approach at the end of the project, we will convene a stakeholder dialogue using the McMaster Health Forum’s approach. The dialogue will provide 18–22 health-system leaders the opportunity to bring their tacit knowledge and their views and experiences to bear on the problem, options, and implementation considerations and, in turn, to learn from the evidence brief and from others’ knowledge, views, and experiences. The stakeholder dialogue will (1) be informed by a pre-circulated evidence brief (as described earlier); (2) be informed by a discussion about the full range of factors that can inform how to approach the problem and possible options for addressing it; (3) bring together multiple stakeholders from across Canada involved in or affected by future decisions related to the issue; (4) ensure fair representation among policymakers, health providers, researchers, and other stakeholders; (5) engage a facilitator from the McMaster Health Forum to assist with deliberations; (6) allow for frank, off-the-record, deliberations by following the Chatham House rule ‘Participants are free to use the information received during the meeting, but neither the identity nor the affiliation of the speaker(s), nor that of any other participant, may be revealed’; and (7) not aim for consensus. Following the dialogue, we will produce a thematic analysis of the deliberations. In addition to these activities, we will publish our findings and present at relevant national and/or international conferences.

## Discussion

This project will advance knowledge and research by synthesizing findings from all of the systematic reviews related to preventing, delaying the onset, or decreasing the burden of frailty-related symptoms and outcomes among older adults. As a result, our project will also contribute to (1) advancing healthcare by allowing for better identification, assessment, and treatment of frail older adults; (2) advancing health systems by supporting the adaptation and implementation of interventions in the Canadian context by convening a stakeholder dialogue with Canadian health-system leaders; and (3) improving health outcomes by ensuring the right mix of programmes, services, and drugs get to those who need them.

## Additional files

Additional file 1:
**Literature search strategy.** Search strategy used to identify literature for the overview of reviews. (DOCX 95.9 kb) Additional file 2:
**Coding framework for conceptual mapping of systematic reviews.** Categories to be used for the conceptual mapping phase of the overview of systematic reviews. (DOCX 19.6 kb)

## Abbreviations

PRESS: Peer Review of Electronic Search Strategies
MeSH: Medical Subject Headings
PRISMA: Preferred Reporting Items for Systematic reviews and Meta-Analyses
AMSTAR: A Measurement Tool to Assess Systematic Reviews

## References

[1] Medline Medical Subject Heading. PubMed 2014 August 14; Available from: URL: http://www.ncbi.nlm.nih.gov/mesh?Db=mesh&term=Frail+Elderly.

[2] Walston J, Hadley EC, Ferrucci L, Guralnik JM, Newman AB, Studenski SA (2006). Research agenda for frailty in older adults: toward a better understanding of physiology and etiology—summary from the American Geriatrics Society/National Institute on Aging Research Conference on Frailty in Older Adults. J Am Geriatr Soc.

[3] de Vries NM, Staal JB, Olde Rikkert MGMNSMWG (2013). Evaluative Frailty Index for Physical Activity (EFIP): a reliable and valid instrument to measure changes in level of frailty. Phys Ther.

[4] Hogan DB, MacKnight C, Bergman H, On behalf of the Steering Committee (2003). Canadian initiative on frailty and aging. Aging.

[5] Fried LP, Walston J, Hazzard WR, Blass JP, Ettinger WJ, Halter JB, Ouslander J (1998). Frailty and failure to thrive. Principles of geriatric medicine and gerontolog.

[6] Fried LP, Tangen CM, Walston J, Newman AB, Hirsch C, Gottdiener J (2001). Frailty in older adults: evidence for a phenotype. J Gerontol A Biol Sci Med Sci.

[7] Inouye SK, Studenski S, Tinetti ME, Kuchel GA (2007). Geriatric syndromes: clinical, research, and policy implications of a core geriatric concept. J Am Geriatr Soc.

[8] Rockwood K, Mitnitski A (2011). Frailty defined by deficit accumulation and geriatric medicine defined by frailty. Clin Geriatr Med.

[9] Sternberg SA, Schwartz AW, Karunananthan S, Bergman H, Mark CA (2011). The identification of frailty: a systematic literature review. J Am Geriatr Soc.

[10] Syddall H, Roberts HC, Evandrou M, Cooper C, Bergman H, Sayer AA (2010). Prevalence and correlates of frailty among community-dwelling older men and women: findings from the Hertfordshire Cohort Study. Age Ageing.

[11] Clegg A, Young J, Iliffe S, Rikkert MO, Rockwood K (2013). Frailty in elderly people. The Lancet.

[12] Walston J, McBurnie M, Newman A (2002). Frailty and activation of the inflammation and coagulation systems with and without clinical comorbidities: Results from the cardiovascular health study. Arch Intern Med.

[13] Puts MTE, Lips P, Deeg DJH (2005). Static and dynamic measures of frailty predicted decline in performance-based and self-reported physical functioning. J Clin Epidemiol.

[14] Lally F, Crome P (2007). Understanding frailty. Postgrad Med J.

[15] Fugate Woods N, LaCroix AZ, Gray SL, Aragaki A, Cochrane BB, Brunner RL (2005). Frailty: emergence and consequences in women aged 65 and older in the Women's health initiative observational study. J Am Geriatr Soc.

[16] Bortz WM (2002). A conceptual framework of frailty: a review. J Gerontol A Biol Sci Med Sci.

[17] Avila-Funes JA, Helmer C, Amieva H, Barberger-Gateau P, Le Goff M, Ritchie K (2008). Frailty among community-dwelling elderly people in France: the three-city study. J Gerontol A Biol Sci Med Sci.

[18] Boyd CM, Xue QL, Simpson CF, Guralnik JM, Fried LP (2005). Frailty, hospitalization, and progression of disability in a cohort of disabled older women. Am J Med.

[19] Pel-Littel RE, Schuurmans MJ, Emmelot-Vonk MH, Verhaar HJJ (2009). Frailty: defining and measuring of a concept. J Nutr Health Aging.

[20] Hoeck S, Francois G, Geerts J, Van der Heyden J, Vandewoude M, Van Hal G (2012). Health-care and home-care utilization among frail elderly persons in Belgium. Eur J Public Health.

[21] Carlson JE, Zocchi KA, Bettencourt DM, Gambrel ML, Freeman JL, Zhang D (1998). Measuring frailty in the hospitalized elderly: concept of functional homeostasis. Am J Phys Med Rehabil.

[22] Rockwood K, Stolee P, McDowell I (1996). Factors associated with institutionalization of older people in Canada: testing a multifactorial definition of frailty. J Am Geriatr Soc.

[23] Rockwood K, Andrew M, Mitnitski A (2007). A comparison of two approaches to measuring frailty in elderly people. J Gerontol A Biol Sci Med Sci.

[24] Ensrud KE, Ewing SK, Taylor BC (2008). Comparison of 2 frailty indexes for prediction of falls, disability, fractures, and death in older women. Arch Intern Med.

[25] Rozzini R, Frisoni GB, Franzoni S, Trabucchi M (2000). Change in functional status during hospitalization in older adults: a geriatric concept of frailty. J Am Geriatr Soc.

[26] Pialoux T, Goyard J, Lesourd B (2012). Screening tools for frailty in primary health care: a systematic review. Geriatr Gerontol Int.

[27] Elkan R, Kendrick D, Dewey M, Hewitt M, Robinson J, Blair M (2001). Effectiveness of home based support for older people: systematic review and meta-analysis. BMJ.

[28] Barlow J, Singh D, Bayer S, Curry R (2007). A systematic review of the benefits of home telecare for frail elderly people and those with long-term conditions. J Telemed Telecare.

[29] Bauer M, Fitzgerald L, Haesler E, Manfrin M (2009). Hospital discharge planning for frail older people and their family. Are we delivering best practice? A review of the evidence. J Clin Nurs.

[30] Chou CH, Hwang CL, Wu YT (2012). Effect of exercise on physical function, daily living activities, and quality of life in the frail older adults: a meta-analysis. Arch Phys Med Rehabil.

[31] Gustafsson S, Edberg A, Johansson B, Dahlin-Ivanoff S (2009). Multi-component health promotion and disease prevention for community-dwelling frail elderly persons: a systematic review. European J Ageing.

[32] Bergman H, Ferrucci L, Guralnik J, Hogan DB, Hummel S, Karunananthan S (2007). Frailty: an emerging research and clinical paradigm—issues and controversies. J Gerontol A Biol Sci Med Sci.

[33] Borges LL, Menezes RL (2011). Definitions and markers of frailty: a systematic review of literature. Rev Clin Gerontol.

[34] Campbell AJ, Buchner DM (1997). Unstable disability and the fluctuations of frailty. Age Ageing.

[35] Daniels R, van Rossum E, de Witte L, Kempen G, van den Heuvel W (2008). Interventions to prevent disability in frail community-dwelling elderly: a systematic review. BMC Health Serv Res.

[36] Morley JE, Perry HM, Miller DK (2002). Editorial: something about frailty. J Gerontol A Biol Sci Med Sci.

[37] Rockwood K, Stadnyk K, MacKnight C, McDowell I, Hébert RA, Hogan DB (1999). A brief clinical instrument to classify frailty in elderly people. Lancet.

[38] Rockwood K (2005). What would make a definition of frailty successful?. Age Ageing.

[39] Sourial N, Wolfson C, Bergman H, Zhu B, Karunananthan S, Quail J (2010). A correspondence analysis revealed frailty deficits aggregate and are multidimensional. J Clin Epidemiol.

[40] Strawbridge WJ, Shema SJ, Balfour JL, Higby HR, Kaplan GA (1998). Antecedents of frailty over three decades in an older cohort. J Gerontol B Psychol Sci Soc Sci.

[41] Sampson M, McGowan J, Lefebvre C, Moher D, Grimshaw J (2008). PRESS: peer review of electronic search strategies.

[42] Lavis JN, Wilson MG, Moat KA, Hammill AC, Boyko JA, Grimshaw JM, Flottorp S. Developing and refining the methods for a ‘one-stop shop’ for research evidence about health systems. Health Res Policy Syst. 2015;13(10). doi:10.1186/1478-4505-13-10 10.1186/1478-4505-13-10 PMC4429608 25971248

[43] Boyd CM, Fortin M (2010). Future of multimorbidity research: how should understanding of multimorbidity inform health system design?. Public Health Rev.

[44] Wagner EH, Austin BT, Von KM (1996). Organizing care for patients with chronic illness. Milbank Quarterly.

[45] Institute for Healthcare Improvement. The IHI Triple Aim. Institute for Healthcare Improvement 2014 March 28;Available from: URL: http://www.ihi.org/Engage/Initiatives/TripleAim/Pages/default.aspx

[46] Shea B, Grimshaw J, Wells G, Boers M, Andersson N, Hamel C (2007). Development of AMSTAR: a measurement tool to assess the methodological quality of systematic reviews. BMC Med Res Methodol.

[47] International Initiative for Impact Evaluation. Evidence gap maps. International Initiative for Impact Evaluation 2014 March 28;Available from: URL: http://www.3ieimpact.org/en/evidence/gapmaps/.

[48] Moher D, Liberati A, Tetzlaff J, Altman DG, The PRISMA Group (2009). Preferred reporting items for systematic reviews and meta-analyses: the PRISMA statement. PLoS Med.

