# Supplementary material for: Interventions for preventing, delaying the onset, or decreasing the burden of frailty: an overview of systematic reviews
Source: Syst Rev. 2015 Sep 25;4:128. doi: 10.1186/s13643-015-0110-7 (PMC4589080; doi:10.1186/s13643-015-0110-7)
Supplement: Additional file 1: — Literature search strategy. Search strategy used to identify literature for the overview of reviews. (DOCX 95.9 kb) [file 13643_2015_110_MOESM1_ESM.docx]

**Additional File 1 – Literature Search Strategy**

**(17 June 2015)**

Search date: 2015Jun17

Limits: None

Alerts: Monthly, when possible

Databases (searched by KC):

- Medline, Embase, HealthSTAR, PsycINFO (Ovid)
- CINAHL, Social Sciences Abstracts, Social Work Abstracts (EBSCO)
- Cochrane Library, including: CDSR, DARE, HTA Database (Wiley)
- EconLit, Social Services Abstracts, Sociological Abstracts, ASSIA - Applied Social Sciences Index and Abstracts (ProQuest)
- LILACS database: <http://lilacs.bvsalud.org/en/>

Additional resources (to be searched by additional team members):

- McMaster Optimal Aging Portal (a repository of reviews and primary research related to optimal aging)
- HealthEvidence.org (for public health related reviews)
- Health Systems Evidence ([www.healthsystemsevidence.org](http://www.healthsystemsevidence.org) a repository of systematic reviews addressing health system topics)
- Open Grey
- Grey Literature Report
- targeted searches of websites/resources such as the Canadian Initiative on Frailty & Aging and the Canadian Geriatrics Society

**Frail Elderly Concept**

Frail Elderly/

Frailty/ use oemezd

(frail* AND (aged OR aging OR elderly OR elder? OR older OR senior?)).tw,kw.

(frail* AND ((geriatric* OR gerontolog*) OR (vulnerable AND older))).mp.

frail*.mp. AND (geriatric* or gerontolog* or aging).jn.

(functional* ADJ2 (declin* OR impair*) ADJ3 (aged OR aging OR elderly OR elder? OR older OR senior?)).tw.

frail*.tw,kw.

limit 7 to "all aged (65 and over)"

limit 7 to "380 aged <age 65 yrs and older>"

limit 7 to aged <65+ years>

**Systematic Reviews/Meta-Analysis/Health Technology Assessment – OVID Medline, EMBASE, PsycINFO** [From: CADTH database search filters [Internet]. Ottawa: Canadian Agency for Drugs and Technologies in Health; 2014. [cited yyyy mm dd]. Available from: [/resources/finding-evidence](https://www.cadth.ca/resources/finding-evidence)]

meta-analysis.pt.

meta-analysis/ or systematic review/ or meta-analysis as topic/ or "meta analysis (topic)"/ or "systematic review (topic)"/ or exp technology assessment, biomedical/

((systematic* adj3 (review* or overview* or search*)) or (methodologic* adj3 (review* or overview*))).ti,ab.

((quantitative adj3 (review* or overview* or synthes*)) or (research adj3 (integrati* or overview*))).ti,ab.

((integrative adj3 (review* or overview*)) or (collaborative adj3 (review* or overview*)) or (pool* adj3 analy*)).ti,ab.

(data synthes* or data extraction* or data abstraction*).ti,ab.

(handsearch* or hand search*).ti,ab.

(mantel haenszel or peto or der simonian or dersimonian or fixed effect* or latin square*).ti,ab.

(met analy* or metanaly* or technology assessment* or HTA or HTAs or technology overview* or technology appraisal*).ti,ab.

(meta regression* or metaregression*).ti,ab.

(meta-analy* or metaanaly* or systematic review* or biomedical technology assessment* or bio-medical technology assessment*).mp,hw.

(grey literature AND review*).tw.

(meta-analysis or systematic review).md.

or/

**Databases:** Ovid HealthSTAR<1966 to May 2015>, Ovid MEDLINE(R) In-Process & Other Non-Indexed Citations and Ovid MEDLINE(R) <1946 to Present>, PsycINFO <1987 to June Week 2 2015>, Embase <1974 to 2015 June 16>

**Search Strategy:**

| **#** | **Searches** | **Results** |
| --- | --- | --- |
| 1 | Frail Elderly/ | 21407 |
| 2 | Frailty/ use oemezd | 246 |
| 3 | (frail* and (aged or aging or elderly or elder? or older or senior?)).tw,kw. | 29696 |
| 4 | (frail* and (geriatric* or gerontolog* or (vulnerable and older))).mp. | 15031 |
| 5 | frail*.mp. and (geriatric* or gerontolog* or aging).jn. | 1320 |
| 6 | (functional* adj2 (declin* or impair*) adj3 (aged or aging or elderly or elder? or older or senior?)).tw. | 2676 |
| 7 | frail*.tw,kw. | 37906 |
| 8 | limit 7 to "all aged (65 and over)" [Limit not valid in PsycINFO,Embase; records were retained] | 32968 |
| 9 | limit 7 to "380 aged <age 65 yrs and older>" [Limit not valid in HealthSTAR,Ovid MEDLINE(R),Ovid MEDLINE(R) In-Process,Embase; records were retained] | 36809 |
| 10 | limit 7 to aged <65+ years> [Limit not valid in HealthSTAR,Ovid MEDLINE(R),Ovid MEDLINE(R) In-Process; records were retained] | 30337 |
| 11 | or/1-6,8-10 | 49490 |
| 12 | meta-analysis.pt. | 116323 |
| 13 | meta-analysis/ or systematic review/ or meta-analysis as topic/ or "meta analysis (topic)"/ or "systematic review (topic)"/ or exp technology assessment, biomedical/ | 347443 |
| 14 | ((systematic* adj3 (review* or overview* or search*)) or (methodologic* adj3 (review* or overview*))).ti,ab. | 276048 |
| 15 | ((quantitative adj3 (review* or overview* or synthes*)) or (research adj3 (integrati* or overview*))).ti,ab. | 24819 |
| 16 | ((integrative adj3 (review* or overview*)) or (collaborative adj3 (review* or overview*)) or (pool* adj3 analy*)).ti,ab. | 44120 |
| 17 | (data synthes* or data extraction* or data abstraction*).ti,ab. | 48257 |
| 18 | (handsearch* or hand search*).ti,ab. | 19616 |
| 19 | (mantel haenszel or peto or der simonian or dersimonian or fixed effect* or latin square*).ti,ab. | 47797 |
| 20 | (met analy* or metanaly* or technology assessment* or HTA or HTAs or technology overview* or technology appraisal*).ti,ab. | 18285 |
| 21 | (meta regression* or metaregression*).ti,ab. | 11304 |
| 22 | (meta-analy* or metaanaly* or systematic review* or biomedical technology assessment* or bio-medical technology assessment*).mp,hw. | 526673 |
| 23 | (grey literature and review*).tw. | 4307 |
| 24 | (meta-analysis or systematic review).md. | 22900 |
| 25 | or/12-24 | 699159 |
| 26 | 11 and 25 | 1620 |
| 27 | remove duplicates from 26 | 804 |

**The Cochrane Library** [Issue 6 of 12, 2015 (CDSR); 2 of 4, 2015 (HTA Database)]

ID Search Hits

#1 MeSH descriptor: [Frail Elderly] this term only 533

#2 frail* and (aged or aging or elderly or elder? or older or senior?):ti,ab,kw or frail* and ((geriatric* or gerontolog*) or (vulnerable and older)) or functional* near/2 (declin* or impair*) near/3 (aged or aging or elderly or elder? or older or senior?):ti,ab,kw (Word variations have been searched) 1312

#3 #1 or #2 1312

CDSR=89

DARE=65

HTA Database=16

**LILACS Database (**[**http://lilacs.bvsalud.org/en/**](http://lilacs.bvsalud.org/en/)**)**

Systematic reviews=5

**CINAHL (EBSCO)**

| **#** | **Query** | **Results** |
| --- | --- | --- |
| S14 | S5 AND S13 | 48 |
| S13 | S6 OR S7 OR S8 OR S9 OR S10 OR S11 OR S12 | 57,823 |
| S12 | TI ( meta-analy* or metaanaly* or systematic review* or biomedical technology assessment* or bio-medical technology assessment* ) OR AB ( meta-analy* or metaanaly* or systematic review* or biomedical technology assessment* or bio-medical technology assessment* ) OR TI ( grey literature and review* ) OR AB ( grey literature and review* ) | 34,129 |
| S11 | TI ( met analy* or metanaly* or technology assessment* or HTA or HTAs or technology overview* or technology appraisal* ) OR AB ( met analy* or metanaly* or technology assessment* or HTA or HTAs or technology overview* or technology appraisal* ) OR TI ( meta regression* or metaregression* ) OR AB ( meta regression* or metaregression* ) | 1,907 |
| S10 | TI ( handsearch* or hand search* ) OR AB ( handsearch* or hand search* ) OR TI ( mantel haenszel or peto or der simonian or dersimonian or fixed effect* or latin square* ) OR AB ( mantel haenszel or peto or der simonian or dersimonian or fixed effect* or latin square* ) | 4,498 |
| S9 | TI ( ((integrative N3 (review* or overview*)) or (collaborative N3 (review* or overview*)) or (pool* N3 analy*)) ) OR AB ( ((integrative N3 (review* or overview*)) or (collaborative N3 (review* or overview*)) or (pool* N3 analy*)) ) OR TI ( data synthes* or data extraction* or data abstraction* ) OR AB ( data synthes* or data extraction* or data abstraction* ) | 7,789 |
| S8 | TI ( ((systematic* N3 (review* or overview* or search*)) or (methodologic* N3 (review* or overview*))) ) OR AB ( ((systematic* N3 (review* or overview* or search*)) or (methodologic* N3 (review* or overview*))) ) OR TI ( ((quantitative N3 (review* or overview* or synthes*)) or (research N3 (integrati* or overview*))) ) OR AB ( ((quantitative N3 (review* or overview* or synthes*)) or (research N3 (integrati* or overview*))) ) | 30,255 |
| S7 | (MH "Systematic Review") | 22,621 |
| S6 | (MH "Meta Analysis") OR (MH "Meta Synthesis") | 16,450 |
| S5 | S1 OR S2 OR S3 OR S4 | 5,939 |
| S4 | TI ( (functional* N2 (declin* or impair*) N3 (aged or aging or elderly or elder? or older or senior?)) ) OR AB ( (functional* N2 (declin* or impair*) N3 (aged or aging or elderly or elder? or older or senior?)) ) | 428 |
| S3 | TX (frail* and (geriatric* or gerontolog* or (vulnerable and older))) | 4,112 |
| S2 | TI ( (frail* and (aged or aging or elderly or elder? or older or senior?)) ) OR AB ( (frail* and (aged or aging or elderly or elder? or older or senior?)) ) | 3,404 |
| S1 | (MH "Frail Elderly") | 3,617 |

**Social Sciences Abstracts, Social Work Abstracts (EBSCO)**

| **#** | **Query** | **Results** |
| --- | --- | --- |
| S12 | S4 AND S11 | 33 |
| S11 | S5 OR S6 OR S7 OR S8 OR S9 OR S10 | 7,591 |
| S10 | TI ( meta-analy* or metaanaly* or systematic review* or biomedical technology assessment* or bio-medical technology assessment* ) OR AB ( meta-analy* or metaanaly* or systematic review* or biomedical technology assessment* or bio-medical technology assessment* ) OR TI ( grey literature and review* ) OR AB ( grey literature and review* ) | 4,107 |
| S9 | TI ( met analy* or metanaly* or technology assessment* or HTA or HTAs or technology overview* or technology appraisal* ) OR AB ( met analy* or metanaly* or technology assessment* or HTA or HTAs or technology overview* or technology appraisal* ) OR TI ( meta regression* or metaregression* ) OR AB ( meta regression* or metaregression* ) | 288 |
| S8 | TI ( handsearch* or hand search* ) OR AB ( handsearch* or hand search* ) OR TI ( mantel haenszel or peto or der simonian or dersimonian or fixed effect* or latin square* ) OR AB ( mantel haenszel or peto or der simonian or dersimonian or fixed effect* or latin square* ) | 1,188 |
| S7 | TI ( ((integrative N3 (review* or overview*)) or (collaborative N3 (review* or overview*)) or (pool* N3 analy*)) ) OR AB ( ((integrative N3 (review* or overview*)) or (collaborative N3 (review* or overview*)) or (pool* N3 analy*)) ) OR TI ( data synthes* or data extraction* or data abstraction* ) OR AB ( data synthes* or data extraction* or data abstraction* ) | 498 |
| S6 | TI ( ((systematic* N3 (review* or overview* or search*)) or (methodologic* N3 (review* or overview*))) ) OR AB ( ((systematic* N3 (review* or overview* or search*)) or (methodologic* N3 (review* or overview*))) ) OR TI ( ((quantitative N3 (review* or overview* or synthes*)) or (research N3 (integrati* or overview*))) ) OR AB ( ((quantitative N3 (review* or overview* or synthes*)) or (research N3 (integrati* or overview*))) ) | 2,791 |
| S5 | SU meta-analysis OR meta-synthesis OR SU Systematic Review | 1,574 |
| S4 | S1 OR S2 OR S3 | 1,713 |
| S3 | TI ( (functional* N2 (declin* or impair*) N3 (aged or aging or elderly or elder? or older or senior?)) ) OR AB ( (functional* N2 (declin* or impair*) N3 (aged or aging or elderly or elder? or older or senior?)) ) | 163 |
| S2 | TI ( (frail* and (aged or aging or elderly or elder? or older or senior?)) ) OR AB ( (frail* and (aged or aging or elderly or elder? or older or senior?)) ) OR TX ( (frail* and (geriatric* or gerontolog* or (vulnerable and older))) ) | 1,537 |
| S1 | SU frail elderly | 344 |

**EconLit [1969-current], Social Services Abstracts [1979-present], Sociological Abstracts [1952-present], ASSIA - Applied Social Sciences Index and Abstracts [1987-current] (ProQuest)**

(TI(frail* and (aged or aging or elderly or elder? or older or senior?)) OR AB(frail* and (aged or aging or elderly or elder? or older or senior?)) OR SU(frail* and (aged or aging or elderly or elder? or older or senior?)) OR ALL(frail* and (geriatric* or gerontolog* or (vulnerable and older))) OR TI(functional* NEAR/2 (declin* or impair*) NEAR/3 (aged or aging or elderly or elder? or older or senior?)) OR AB(functional* NEAR/2 (declin* or impair*) NEAR/3 (aged or aging or elderly or elder? or older or senior?)) OR SU(functional* NEAR/2 (declin* or impair*) NEAR/3 (aged or aging or elderly or elder? or older or senior?))) AND (TI(meta-analy* OR metaanaly* OR metanaly* OR "technology assessment" OR HTA OR HTAs OR "technology overview" OR "technology appraisal" OR "meta regression" OR metaregression* OR systematic review* OR systematic search* OR "grey literature") OR AB(meta-analy* OR metaanaly* OR metanaly* OR "technology assessment" OR HTA OR HTAs OR "technology overview" OR "technology appraisal" OR "meta regression" OR metaregression* OR systematic review* OR systematic search* OR "grey literature") OR SU(meta-analy* OR metaanaly* OR metanaly* OR "technology assessment" OR HTA OR HTAs OR "technology overview" OR "technology appraisal" OR "meta regression" OR metaregression* OR systematic review* OR systematic search* OR "grey literature"))

=37 results
