# Supplementary material for: Interventions for preventing, delaying the onset, or decreasing the burden of frailty: an overview of systematic reviews
Source: Syst Rev. 2015 Sep 25;4:128. doi: 10.1186/s13643-015-0110-7 (PMC4589080; doi:10.1186/s13643-015-0110-7)
Supplement: Additional file 2: — Coding framework for conceptual mapping of systematic reviews. Categories to be used for the conceptual mapping phase of the overview of systematic reviews. (DOCX 19.6 kb) [file 13643_2015_110_MOESM2_ESM.docx]

**Additional file 2 – Coding framework for conceptual mapping of systematic reviews**

| **Coding domain** |  |
| --- | --- |
| Type of sector(s) involved | - Primary care - Home care - Hospital care - Rehabilitation - Long-term care - Public health |
| Type of provider(s) involved | - Physician   - Generalist   - Specialist     - Geriatrician - Nurse - Nurse practitioner - Pharmacist - Allied health professional - Lay/community health worker |
| Type of intervention(s) evaluated* | - **Preventing frailty**   - Identifying those at risk   - Health promotion   - Other (specify)*: - **Delaying the onset of frailty**   - Physical exercise   - Occupational therapy   - Home-based care   - Other (specify)*: - **Decreasing the burden of frailty-related symptoms (based on six domains for the Chronic Care Model)**   - self-management support (e.g., providing information, emotional support and strategies for living with chronic health conditions)   - decision support (e.g., encouraging the use of medical guidelines and tools to help patients make decisions)   - delivery of care (e.g., home-based care/support, case management, providing culturally-sensitive care, team-based care, hospital discharge planning)   - clinical information systems (e.g., electronic health records that provide reminders for providers and patients, and monitors the performance of healthcare teams and the broader health system)   - health system changes (e.g., agreements to facilitate care coordination within and across organizations)   - community resources and policies (e.g., forming partnerships with community organizations to fill gaps in services)   - Other (specify)*: |
| Types of outcomes included in the analysis | - Improving the patient experience of care   - access to and availability of care   - appropriateness of care and quality of care   - patient satisfaction - Improving the health of populations   - disease-focused outcomes   - health-related quality of life   - patient goals met and mortality - Reducing the per capita cost of care   - costs associated with the intervention   - formal economic evaluation (e.g., cost-effectiveness analysis) |

*Categories to revised and supplemented based on groups of interventions noted during the title and abstract review phase.
